# Mr. Jonathan Hutchinson, F.R.S.

**Published:** 1903-08-01

**Authors:** 


### The Hospital.

A Journal of

The Medical Sciences and Hospital Administration.

Vol. XXXIV.—No. 879. Edited by Sir Henry Burdett, K.C.B. [August 1, 1903.

### Mr. Jonathan Hutchinson, F.R.S.

It was fitting that Mr. Jonathan Hutchinson at the end of 50 years' practice on his seventy-fifth birthday should be entertained to dinner by some 200 members of the medical profession. We noted with regret the absence of a number of men, old and former colleagues and others, whose personal indebtedness to Mr. Hutchinson must be great indeed. The gathering on the 23rd inst. was, however, widely representative of much that is best in the fields of work which Mr. Hutchinson has cultivated so assiduously during a long and busy life. As Sir Henry Howse well put it, the variety and extent of Mr. Hutchinson's life-work must force the historian to begin at Genesis and end at Revelation, but happily we are not yet at the end of his useful labours. Replying to the toast of his health Mr. Hutchinson wittily referred to a member of the profession who, his contemporaries declared, was ruined in life by believing his own testimonials. He hoped to avoid that rock, and would simply state that his constant aim through life had been fidelity to conscience. He added that a man's life work might bring him fame by handing his name down to all time, but that the true test of the value of such work was that the man's acts and his influence upon his contemporaries should be such that they shaped the course of events and so secured him real immortality on earth. All who have a personal knowledge of Mr. Hutchinson, of his great erudition, his extended reading, his marvellous memory, his power to collect, assimilate, and group facts which has made him one of the most successful of teachers, his charm as a host, his true neighbourliness and keen interest in everything affecting the welfare of the district where his country home is situated, recognise that these gifts have conferred upon Mr. Hutchinson unusual distinction, as they have won for him the affection of his fellows.

Of Mr. Hutchinson's early career and work little need be said. He received his professional education at St. Bartholomew's Hospital, became a fellow of the College of Surgeons in 1862, has filled all the principal offices of the college, including those of Hunterian Professor of Surgery and Pathology 1878–1883, member of Council 1879–1895, examiner 1880–1887, vice-president 1886, Bradshaw lecturer 1888, president 1889, and finally Hunterian orator in 1891. From 1859 to 1883 he was surgeon to the London Hospital, and since the latter date has been consulting surgeon to that charity.

It is difficult on first thoughts to refer Mr. Hutchinson to his proper position in the profession, for he has worked hard and with singular success in many different branches. One regards him primarily as a great syphilographer, for "Hutchinson's teeth" are perhaps destined to make his name better known, and for a longer time than any of his other work. His clinical labours in general surgery, his reputation as an ophthalmic surgeon, and his skill as a dermatologist are all of the highest order. But his teaching powers are no less remarkable than his powers of observation. The mainstay of the post-graduate teaching in London and Emeritus professor at the London Hospital, his less gifted neighbours at Haslemere and Selby have discovered that he is able to teach them geology and field natural history, and to this end he has founded educational museums from which they may reap the greatest advantage. Like John Hunter, Sir James Paget and Sir George Humphry, Mr. Hutchinson has the instinct of collecting very markedly developed, and with it the power of classifying and arranging his collections so that everything may be in order and the relationship of one specimen to another may be duly exhibited. This power of collecting, observing, and comparing has made him serviceable to the whole profession of medicine, for it has enabled him to produce the illustrations of "Clinical Surgery" and the "Archives of Surgery," both publications of first-rate importance. Yet, with all this, his energies are not exhausted, for the burden of managing the Sydenham Society has rested upon his shoulders for many years, and for a time he was editor of the *British Medical Journal*. The simplicity of his life, his entire want of ostentation, and his extreme hospitality attest his Quaker ancestry, whilst as a man of business and a landlord his north-country shrewdness has stood him in very good stead. But with all these claims to recognition there is no doubt that he would prefer to be remembered as a great thinker.

English surgery has but few men just now with original ideas: many can operate and more still can make money by their art, which they look upon rather as a trade than a profession. But the power of enunciating new truths remains, as it always has been, a rare gift. Surgical truths do not lie open to the first comer, but have often to be sought patiently and by a process of generalisation from groups of cases which too often present all kinds of fallacies and pitfalls to the unskilled interpreter. It is exactly in the interpretation of these cases that Mr. Jonathan Hutchinson has shown such marked success, and the advance of years has not rendered him less observant or less enthusiastic in his collection of all that is rare and valuable in clinical surgery. Last week he used the opportunity of an after-dinner speech to impress upon his audience the necessity of making careful notes, and if possible, drawings, of any rare cases which might come under their notice. It is remarkable that such a career should not have secured, as it has undoubtedly entitled Mr. Hutchinson to receive, the honour at least of a baronetcy.

